# Evaluating robotic-assisted partial nephrectomy surgeons with fully convolutional segmentation and multi-task attention networks

**DOI:** 10.1007/s11701-023-01657-0

**Published:** 2023-06-27

**Authors:** Yihao Wang, Zhongjie Wu, Jessica Dai, Tara N. Morgan, Alaina Garbens, Hal Kominsky, Jeffrey Gahan, Eric C. Larson

**Affiliations:** 1 Department of Computer Science, Southern Methodist University, Dallas, USA; 2 Department of Urology, University of Texas Southwestern Medical Center, Dallas, USA

**Keywords:** Surgical assessment, Convolutional network, Self-attention, Multi-task learning

## Abstract

We use machine learning to evaluate surgical skill from videos during the tumor resection and renography steps of a robotic assisted partial nephrectomy (RAPN). This expands previous work using synthetic tissue to include actual surgeries. We investigate cascaded neural networks for predicting surgical proficiency scores (OSATS and GEARS) from RAPN videos recorded from the DaVinci system. The semantic segmentation task generates a mask and tracks the various surgical instruments. The movements from the instruments found via semantic segmentation are processed by a scoring network that regresses (predicts) GEARS and OSATS scoring for each subcategory. Overall, the model performs well for many subcategories such as force sensitivity and knowledge of instruments of GEARS and OSATS scoring, but can suffer from false positives and negatives that would not be expected of human raters. This is mainly attributed to limited training data variability and sparsity.

## Introduction

The primary objective of this research was to predict, based on video review, surgeon technical performance during the tumor resection and renography steps of actual robotic-assisted partial nephrectomy (RAPN) [1]. Specifically, we develop a machine learning architecture consisting of a multi-task convolutional neural network (mtCNN) [2, 3] that assesses surgeon technical performance using two validated assessment tools: the Objective Structured Assessment of Technical Skills (OSATS) [4] and the Global Evaluative Assessment of Robotic Skills (GEARS) [5]. The developed model provides scoring for each subcategory of the OSATS and GEARS scales. We highlight three specific contributions of this work:

1. We generate a collection of segmented videos scored by human raters for RAPN. Human scores were analyzed for inter-rater matching accuracy of GEARS and OSATS.
2. We modify previous work on synthetic tissue to analyze actual surgeries [6] and expand on our instrument tracking methods to include precise masks of instruments, rather than bounding boxes. Masking performance is also evaluated.
3. We further expand previous work [6] to include prediction of OSATS scores and GEARS scores together from a shared neural network representation. We evaluate this performance compared to human scoring.

The neural network architecture employed here used mechanisms for weighting different portions of the video—this is an automated process known as attention in neural networks and is important when understanding if a segment is in fact “real-world” impact full and not an aberrant signal [7]. We investigate attention using three different mechanisms to identify the most important segments in the procedures for predicting the scores. The three attention mechanisms used were selected based upon previous works in speech processing [8].

## Related work

We divide related work into subcategories including robotic surgery deep learning methods, semantic segmentation, and multi-task learning.

*Robotic surgery using deep learning:* Many previous studies have validated the GEARS and OSATS assessment tools [4, 5], and a number of studies have attempted automated methods for predicting these scales. We limit our discussion here to the most similar methods to ours—those employing deep neural networks for prediction of surgical expertise. Zhao et al. used 2D convolutional networks for tracking surgical instruments for verification [9]. Law et al. employ hourglass networks to locate specific parts of a robotic instrument, using machine learning to make predictions of GEARS scores on two levels (low vs. high) [10]. Lee et al. used instrument tracking with convolutional networks to classify GEARS into three levels. They report accuracy ranging from 57 to 83% for classification [11]. Gahan et al. focused on using convolutional sequence models to assess GEARS scores with up to 78% accuracy among five subcategories [12]. Wang et al. improved upon these methods [6], classifying the GEARS score, and the subcategories of GEARS scores, in its entire dynamic range on synthetic tissue for surgeons in training. Wang et al. employed convolutional networks, achieving good performance with scores matching manual inspection in 86.1% of all GEARS subcategories. While this work showed that evaluation of GEARS subcategories with artificial neural networks is possible for novice and intermediate surgeons, it was unclear if expert surgeons could be evaluated with a similar automated system. This was due to a limited amount of data on expert surgeons in the data set. In this work, we work almost exclusively with individuals that are rated as expert surgeons. Moreover, we further improve upon previous works by introducing segmentation methods and further develop methods to work with videos of real surgery (as opposed to synthetic tissue). We also employ an additional task, regressing the OSATS score, for training the machine learning network.

**Fig. 1:**
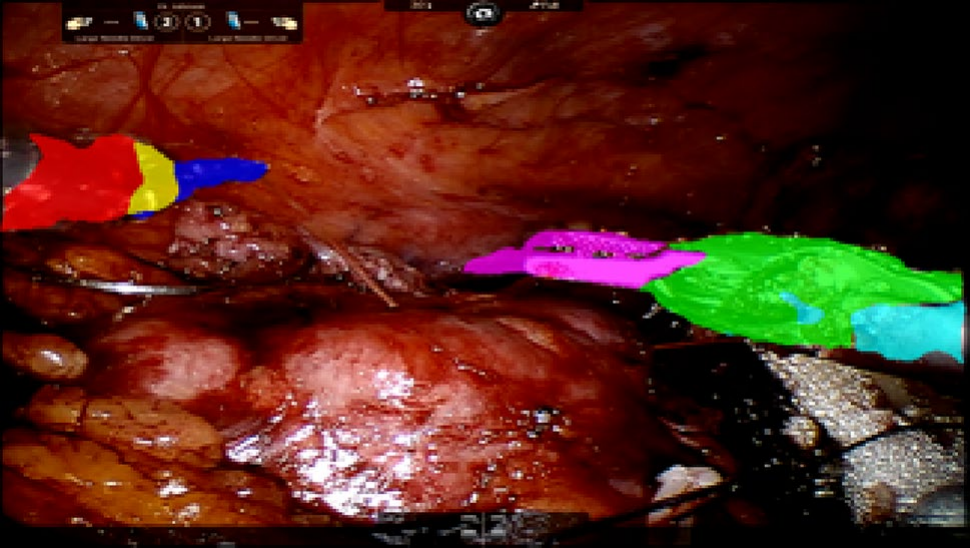
An example segmentation mask used in our collected data set

*Semantic segmentation*: is a task that generates an image mask based on semantic regions. That is, a pixel for pixel mask is generated for each object of interest (see Fig. 1). For example, Garcia et al. and Wang et al. combined semantic segmentation with deep learning on different real-world image data [13, 14]. Marullo et al. used semantic segmentation of instruments in a laparoscopic surgery to classify blood accumulation events with accuracy of 90% [15]. In their approach the main aim was to remove all instruments from the video scene, rather than identify each portion of the instrument with a mask. This motivated us to use these segmentation masks in our work, replacing the key-point detection algorithm from previous work [6]. In the current work, we find that segmentation masks provide additional information by providing knowledge of instrument area (not just location) which can be fed into the model and increase accuracy while decreasing the false positive detection rate of similar instruments (e.g., suction device).

*Multi-task learning*: is a method for exploiting information in a single modeling framework with multiple classification tasks. It has been shown to increase accuracy and generalization in many applications [16]. Typically, this involves training a neural network with two or more labels or loss functions that can be optimized simultaneously. For example, labeled examples from the GEARS and OSATS scoring can be used to simultaneously update the trained parameters of a neural network. In this way, the neural network must learn features that help to discern rating for both the GEARS and OSATS scores. Finally, we also build from previous works that use multiple loss functions for multi-task learning [8]. Cross entropy (CE) is used in previous work as loss function for each GEARS score [6]. However, this ignores the relative difference between actual and predicted scores. For example, CE does not discern that, if the actual score is 5, it is better to predict a 4 than a 3. Therefore, we also conducted experiments with a loss function that takes into account this difference: the weighted-$\kappa$ Loss [17].

## Methods

To inform the design of our networks and evaluate their performance, we developed a dataset of RAPN segmented surgical videos and recruited reviewers to score each segmented video. Surgical assessment has been extensively evaluated for RAPN, as indicated by [18]. Fellows and attending surgeons provided GEARS and OSATS scores based on video review of RAPN surgeries. Reviewers were trained in the OSATS and GEARS systems to score surgery videos. The analysis of de-identified videos was approved by the University institutional review board (IRB). In total, five trained surgeons were able to annotate and score the segmented videos. In addition, the annotators were asked to identify the surgical videos according to the major tasks involved: (1) resection, (2) renography, and (3) placement of bolsters. In each video, two surgeons (one trainee and one attending surgeon) complete each surgical task and were graded separately. It is important to note that the videos were not divided based on performance but were divided between trainee and attending surgeon and the reviewers were not aware of expertise level, only that it was a different surgeon. In instances when a segmented video contained more than a single surgeon, the scores are labeled as “invalid” by reviewers. This may occur because the duration for that surgeon may not be long enough to accurately assess GEARS and OSATS. A summary of the dataset is given in Table 1.

**Table 1:** Summary of collected data

Videos	150
Unique surgeries	50
Annotators/raters	5
Surgeries with multiple raters	10 (20%)
Surgeons per task, cut	Two surgeons: 29, One surgeon: 21
Surgeons per task, recon	Two surgeons: 32, One surgeon: 18
Surgeons per task, bolster	Two surgeons: 40, One surgeon: 10
Video duration, cut	Mean: 12.5 mins (5.1$-$25.0 mins)
Video duration, recon	Mean: 17.4 mins (7.7$-$33.7 mins)
Video duration, bolster	Mean: 10.2 mins (4.4$-$18.5 mins)
Total size, compressed	343 GB

### Machine learning methods

The processing pipeline of our automated scoring system contains several steps related to loading and transforming videos of surgery, segmenting instruments, and scoring the movements of the instruments via GEARS and OSATS regression. An overview of our method is as follows: First, a custom video loading data processor is used to decompress time segments of videos. Second, the object detector is used to identify masks of surgical instruments in each video frame. This is also referred to as semantic segmentation because it segments certain parts of the image that we have labeled as “semantically” meaningful. Third, the coordinates and pixel-area of surgical instruments are calculated and saved to be used as features. Finally, these features are combined over time and used as inputs to train a sequence scoring model. This model takes a variable length set of feature vectors and regresses/classifies to three different outputs: GEARS, OSATS, and the surgical task. We refer to this as the scoring network [2, 3].

*Semantic segmentation*: To train the semantic segmentation network, manual labels are needed. A human reviewer was hired to create masks for each portion of the robotic instrument in a subset of video frames. These labels are used to train our semantic segmentation network. When labeling the objects in the frame, we chose to label the instruments similarly to our previous work, where each movable structure on the robotic instrument is labeled separately [6]. Seven different portions of the instruments (and background) are segmented in the frame as: (1) Left-arm (upper flexion), (2) Left-arm (abduction), (3) Left grasping or cutting, (4) Right-arm (upper flexion), (5) Right-arm (abduction), (6) Right grasping or cutting, (7) Needle (see Fig. 1). Any region that does not belong to the object listed above is assigned to be “background.” This background tag also includes other instruments that are not under the direct control of the surgeon, such as the suction arm.

The structure of the semantic segmentation network is similar to the U-net architecture employed in [6]. The U-net architecture [19] used is a convolutional neural network that contains five encoding blocks, five decoding blocks, and three bottleneck blocks. The real-world nature of these videos adds complexity to the analysis compared to our previous approaches with training videos on synthetic tissue. Therefore, we altered the network from [6] with an image global attention layer to reduce the network’s complexity, resulting in a parameter reduction in the deep network by 45% [20]. In several decoding blocks, we also use cross image attention [21], allowing the network to weight certain portions of the input feature maps differently in the convolutional processing layers.

*Scoring network:* The input to the scoring network is from the output of the semantic segmentation, aggregated over time. Each feature vector can contain up to seven mask objects with three components: the *x* and *y* coordinate of the mask’s center and the mask’s area. Thus, each frame has 21 features (7 for *x*, 7 for *y*, and 7 for area). The scoring network regresses multiple frames into GEARS and OSATS scores. Specifically, the scoring network is tasked with regressing the each subcategory of the GEARS and OSATS scoring, as well as classifying the main task segments of the surgery (e.g., cut, bolster). The scoring network uses a common representation in early layers, but then branches its representations into specifics outputs for each task—thus, it is a multi-task network. Because each task is performed from this common representation and the network employs convolutions across time, the scoring network referred to as *multi-task convolutional neural network* (mtCNN) [3].

**Fig. 2:**
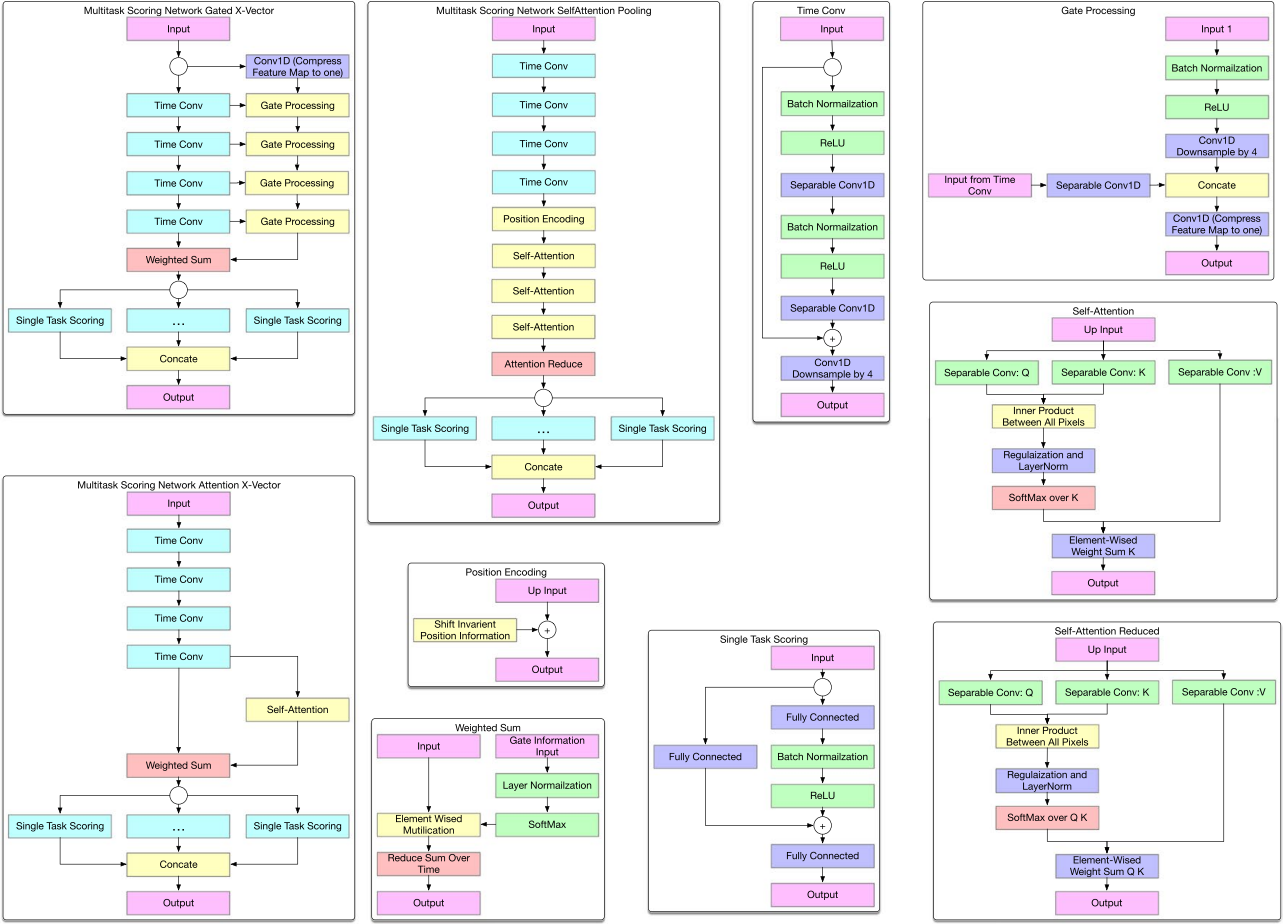
Overview of the mtCNN scoring network

As shown in Fig. 2, the mtCNN processes the sequence of instrument features across all the video frames. Similar to [6], two pathways are traversed in the model, a time convolution path and an attention-based pathway that weights the convolutional outputs. After convolutions are applied and weighted according to attention, they are collapsed using the average of each weighted convolutional filter. This average allows the output to be a consistent length regardless of how long the sequence lasted. We investigate three different methods for the secondary attention pathway. Specifically, we investigate three methods of attention, similar to [8]: (1) Weight Gated, (2) Self-Attention, and (3) Direct Self-Attention Pooling [22, 23]. Each method is shown in Fig. 2 in a separate titled block. Intuitively, each attention method differs in how it tries to generate the weights before taking an average. *Weight Gated* processing uses multiple 1D convolutions followed by a softmax layer to force the network to focus on particular time segments before averaging. *Self-Attention* is used to calculate a set of weight vectors, similar to the use of transformers [7]. *Direct self-attention pooling* uses convolutional self-attention instead of temporal pooling. We investigate which method of attention weighting has the best performance in the context of robotic surgery assessment.

The outputs of the mtCNN are three sets of classifications/regressions: six GEARS subcategories, seven OSATS subcategories, and the type of overall task the surgeon performs (i.e., cut). The result must be compared to human scoring through a loss function to optimize each model. We investigate two loss functions: Cross-Entropy Loss (CE) and Weighted-$\kappa$ Loss (WK) [17]. We adapt the weighted $\kappa$ for use as a loss function. We first use logarithms to decouple the numerator and denominator, simplifying the computation of the gradient [17]. Thus, the problem is reformulated to minimize:

1 $$\begin{aligned}{} & {} l_{WK} = \log (1-\kappa +\epsilon ), \text { where } \kappa =1-\frac{\sum _{i,j}\ w_{i,j}O_{i,j}}{\sum _{i,j} w_{i,j}E_{i,j}} \, \text { and } w_{i,j}=\frac{{(i-j)}^k}{{(N-1)}^k}, \end{aligned}$$

where $\epsilon$ avoids calculating $\log (0)$ and *O*, *w*, and *E* are the matrices of observed scores, penalty weights, and expected scores. $O_{i,j}$ corresponds to the number of surgeries that receive a score *i* from one rater and score *j* from another rater. Matrix *E* is calculated via the outer product between the score vectors of the two raters, normalized to have the same sum as Matrix *O*. In calculating the weight entries, $w_{i,j}$: *N* is the number of possible ratings (in this study $N=5$), *k* indicates the strength of the penalty (we use $k=1$). $\kappa =1$ indicates perfect agreement and $\kappa =0$ indicates no agreement.

## Results

### Semantic segmentation

We employ 872 labeled image frames for training, comprising more than 4,000 labeled pixel masks for the various instruments. We also use several negative examples (images without any surgical instruments) to help reduce false positives.

**Fig. 3:**
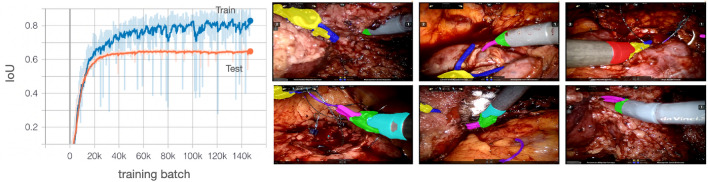
Left: training and validation curves of the semantic segmentation network. Right: a few examples of the segmentation results

The training and evaluation progress of the network is shown in Fig. 3 (left). The y-axis shows the intersection over the union (IoU) of the manually labeled masks and the predicted masks for each batch. IoU is a standard method for assessing the goodness of fit for a segmentation network. It measures the degree of overlap between actual and predicted masks. The x-axis of the figure shows the number of training batches that the network uses for optimization—about 150,000 batches are used, comprising 2,800 epochs (an epoch is one pass through all training data). For evaluation, about 500 labeled masks (not in the training set) are plotted in orange in Fig. 3 (left). During training, the data are randomly scaled, rotated, and shifted using a projection transform, which helps to mitigate overfitting [24]. Evaluation data are not augmented. The training data IoU converge to about 0.8 and the evaluation data converge to an IoU of 0.65, which is similar in performance to other semantic segmentation algorithms [25].

We then tested the semantic segmentation model on a video that was not in the training set. We visually observed that the model had relatively good detection, but improvements were necessary to reduce false positives. Therefore, a series of “enhancement” frames were selected from the video that had many false positives. Labeling was provided for these scenarios to enhance performance. A direct reporting of the IoU from this evaluation video is not possible because manual labeling of each frame of the video is extremely time consuming. We observed that this noticeably reduced false alarms in visual inspections as shown in the examples in Fig. 3 (right). We also observe that the masking of surgical instrument parts is quite robust. In some cases, it is difficult even for the human reviewer to identify the different portions of the instruments because of various angles, extreme positions, and obstruction by tissue/blood. The most common errors we observe are boundary errors where the mask is slightly too large or small at the instrument edges. The result could be further improved by extracting even more frames from the video—however, we have found a perfect tracking result is not required to obtain good performance in GEARS and OSATS prediction and some “noise” from the semantic segmentation network can be tolerated and accounted for by the scoring network.

### Architecture and loss function investigation

Most GEARS and OSATS subcategory scores are either rated three–five demonstrating an imbalance in the data collected. The vast majority of the data is related to expert surgeons. 98% of the scores are designated “expert;” there are no novice scores; and only 2% of the surgery scores fall into the “intermediate” designation. Another possible confound in the data is that the GEARS and OSATS scores are rated for long time segments (about 10 min of maneuvers) [4, 5]. Thus, though the overall score is high, arbitrary time segments within the surgery may be poorer. This may cause some automation problems, such as difficulty in training the model to recognize “poor” intervals.

We evaluated the mtCNN with several competing architecture structures and training loss functions, as previously described. A single training and testing split is used in this analysis, where 80% of surgeries are used for training and 20% for evaluation. We summarize the different overall GEARS and OSATS scores in Table 2. We report the best performing models for training the networks by monitoring performance on the evaluation data and checkpointing when the model surpasses previous performances. Because a single split of the dataset could potentially bias our conclusions, we only use this split to select the loss function and architecture. We do not solely rely on this split to bound performance. However, later analyses that use repeated splits will reveal that the data in Table 2 is representative of overall performance.

**Table 2:** Accuracy of different models using loss functions: cross entropy (CE) or weighted $\kappa$ loss and different attention mechanisms: Weight Gated (WG), Self-Attention (SA), and Direct self-attention Pooling (DP). Bootstrap results are also given for the mean ± standard deviation

	Loss		Attention			GEARS	OSATS	Task
Model	$\kappa$	CE	WG	DP	SA	Acc.	Acc.	Acc.
Rater–Rater	–	–	–	–	–	0.71	0.75	N/A
WG-$\kappa$	$\checkmark$	–	$\checkmark$	–	–	0.35	0.32	0.80
DP-$\kappa$	$\checkmark$	–	–	$\checkmark$	–	0.46	0.61	0.66
SA-$\kappa$	$\checkmark$	–	–	–	$\checkmark$	0.45	0.55	0.91
WG-CE	–	$\checkmark$	$\checkmark$	–	–	0.57	0.59	0.81
DP-CE	–	$\checkmark$	–	$\checkmark$	–	0.52	0.60	0.68
SA-CE	–	$\checkmark$	–	–	$\checkmark$	**0.60**	**0.63**	**0.93**
SA-CE	Bootstrapping Aggregation					$0.59\pm 0.13$	$0.62\pm 0.12$	$0.75\pm 0.22$

Bold in the table indicates the best performing model in each category

Table 2 shows the exact matching accuracy for each attention mechanism and each loss function investigated. The inter-rater exact matching accuracy is also shown on a subset of videos from which two raters scored the same surgery (in the top row). Matching accuracy is calculated by the number of subcategories in GEARS and OSATS that match exactly between two raters (or between model and raters) on a scale of 1–5 for each subcategory. Many of the architectures exhibit similar, but slightly reduced, matching agreement with the human raters. Therefore, there is still room to improve the models. However, a clear best performing model emerges: the self-attention architecture with cross entropy loss (SA-CE).

### GEARS and OSATS bootstrap analysis

While the SA-CE model tends to perform the best on a single split of the data, we also wish to characterize this performance of the model using different training and testing samples, which can be achieved using a bootstrapping sampling method [26]. We trained the SA-CE model repeatedly (over 1000 times) with randomly sampled training data from the dataset. Any data not chosen for training samples is used for evaluation of that training run. For each round, we train the data with augmentation (as described) but evaluate without augmentation. The 0.632 bootstrap results are reported in the bottom row of Table 2. In bootstrapping, evaluation criteria are calculated per round to help bound how the model will perform with a varying set of training and testing examples. Therefore, the average of bootstrapping performance is a good indicator of expected performance on unseen data and the range indicates the highest and lowest possible performances. The mean results are similar to the performance results from our previous analysis with a single split of the data, which indicates these previous results are a good proxy of overall performance. The overall performance is calculated by 0.632 rule [26]. The performance captured with this rule can be considered as the theoretical “actual performance” when the training dataset performance is similar to the evaluation performance. The 0.632 rule does not guarantee this behavior—but we do observe this in our dataset. Thus, we have high confidence the result is not biased. We also note that the peak performances of a number of models using the bootstrap analysis are encouraging, with matching accuracy surpassing human agreement. The maximum performance of any model from the bootstrap was 0.85 for both GEARS and OSATS, which is an optimistic measure of performance. On average, we expect a match of 0.60 or 0.63, compared to human rater matching of 0.71 or 0.75.

**Fig. 4:**
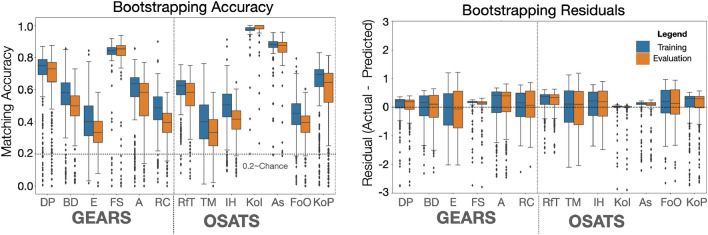
Subcategory matching accuracy and residuals from bootstrap analysis. Subcategories include: Depth Perception (DP), Bimanual Dexterity (BD), Efficiency (E), Force Sensitivity (FS), Autonomy (A), Robotic Control (RC), Respect for Tissue (RfT), Time and Motion (TM), Instrument Handling (IH), Knowledge of Instruments (KoI), Assistance (As), Flow of Operation (FoO), and Knowledge of Procedure (KoP)

We also report the bootstrap matching accuracy for each subcategory of GEARS and OSATS in Fig. 4 (left). The box plot shows the distribution of accuracy on each subcategory for both the training sets and evaluation sets. We see a similar trend with the training and evaluation scores similarly distributed, which provides additional confidence in the bootstrap methodology. Thus, this agreement also extends to each subcategory, not just the overall scores.

For GEARS, we note that the best performing subcategory is “force sensitivity,” while the lowest performing subcategory is “efficiency.” All subcategories are better than chance, but none are exceptionally high scoring. For OSATS, the best performers are “knowledge of instruments” and “assistance,” while the lowest performer is “time and motion.” In OSATS, the best performers have substantially high accuracy, but both of these subcategories have limited variability in the dataset, with most surgeons scoring highly. As such, the results for these scales might be optimistic.

While the distributions in the Fig. 4 (left) show similarity of outputs, they hide raw differences in the scores. As such, it is not clear if the model over or under estimates the GEARS and OSATS ratings. To elucidate this, we report the residual difference between predicted GEARS and OSATS subcategories in Fig. 4 (right). Ideally, each plot would be centered on 0.0 with small interquartile ranges. Because the median values are mostly positive, we observe that the models tend to predict higher scores (i.e., higher than actual skill). We note that this bias is only slight and it is encouraging to see that the median is near zero for all subcategories. This supports a conclusion that the models are not overly producing false negatives or false positives. However, there also appear to be a number of outlier points where some bootstrap models predict a lower skill than actual, as given by the black dots shown in Fig. 4 (right). These outliers show that sometimes the bootstrap models have residuals as large as 3.0 (for some scales). This kind of disagreement was not observed among human raters. On average, the interquartile range is less than 0.5 residual difference, indicating that most subcategories are exact or within one point of the human rater. This conclusion is somewhat limited by the observed variability in our ground truth data.

## Conclusion

We developed a deep learning algorithm for scoring GEARS and OSATS from a dataset robotic partial nephrectomy procedure videos. We found that our network trained with self-attention and cross entropy loss (SA-CE) to perform substantially better than other architectures. Based on our results, we conclude that our methods for automated scoring are substantially better than chance, with some predictions similar in agreement between two surgeon raters. However, some of the automated scores are less reliable than those provided by a skilled surgeon reviewer, requiring further data collection and investigation. Furthermore, because the results are evaluated upon real surgeries, there is limited variability in the scores—most surgeons are experts, scoring highly on all subcategories of OSATS and GEARS. While we conducted our experiments using Intuitive platforms, the only data employed from the platform was the video feed. No other internal calibration or 3D motion data was used from the platform. Therefore, the mtCNN model should generalize to any robotic platform that allows access to video of the surgery being performed. Furthermore, the mtCNN model, once trained, can process video faster than real time. Practically, this also means that a single server could process video from multiple surgical platforms simultaneously. Thus, a single server model could be employed without sacrificing processing time.

## Data Availability

Videos available upon request, with proper data management plan and IRB reciprocity approval.

## Code availability

All source code is custom developed and available upon request.
